# Lunar Phases and Emergency Department Visits for Renal Colic Due to Ureteral Calculus

**DOI:** 10.1371/journal.pone.0157589

**Published:** 2016-06-14

**Authors:** Andy W. Yang, Justin D. Johnson, Carolyn M. Fronczak, Chad A. LaGrange

**Affiliations:** Department of Surgery, Division of Urologic Surgery, University of Nebraska Medical Center, Omaha, Nebraska, United States of America; University of Texas Southwestern Medical Center, UNITED STATES

## Abstract

**Background:**

Urolithiasis affects an estimated 5% of the population and the lifetime risk of passing a stone in the urinary tract is estimated to be 8–10%. Urinary calculus formation is highly variable and while certain risk factors such as age, gender, seasonality, anatomic abnormality, and metabolic diseases have been identified, not much is known regarding the association of environmental factors such as lunar phases on renal colic. We conducted a retrospective study to test the hypothesis that full moon phase is an environmental factor associated for increased emergency department (ED) visits for renal colic due to ureteral calculus.

**Methods:**

We analyzed 559 renal colic diagnoses by the ED at the University of Nebraska Medical Center in a 24-month period and compared them with corresponding lunar phases as well as supermoon events. The lunar phases were defined as full moon ± two days, new moon ± two days, and the days in-between as normal days according to the lunar calendar. Supermoon event dates were obtained from NASA.

**Results:**

90 cases (16.1%) were diagnosed during full moon phase, 89 cases (15.9%) were diagnosed during new moon phase, and 380 cases (68.0%) were diagnosed during normal days. The incidence of renal colic showed no statistically significant association with lunar phases or supermoon events.

**Conclusion:**

In this retrospective longitudinal study with adequate power, neither full moon phase nor supermoon event exhibited an association with increased renal colic diagnoses due to ureteral calculus by the ED at the University of Nebraska Medical Center.

## Introduction

Urolithiasis affects an estimated 5% of the population and the lifetime risk of passing a stone in the urinary tract is estimated to be 8–10% [1]. Urinary calculus formation is highly variable and a specific factor responsible cannot be identified in most patients. While age, gender, seasonality, anatomic abnormalities such as ureteropelvic junction obstruction, and metabolic risk factors such as diabetes mellitus type II have been reported [2–4], many other pathophysiologic, metabolic, and genetic factors responsible for urolithiasis are currently being examined. However, little is known about the association of environmental factors such as lunar phases on ureteral calculus resulting in renal colic.

Human physiology and behavior have been shown to be affected by environmental factors such as seasons and circadian rhythm [5–8]. Lunar phases also have been documented to exhibit an association on human physiology including an increase in seizures [9], aggressive behavior [10], mood disorders [11], changes in menstruation cycle [12], inducing full-term deliveries [13], and even increase in myocardial infarctions [14].

Specifically for the effect of lunar phases on human genitourinary system, two studies thus far have investigated the association between lunar phases and incidence of renal colic due to ureteral calculus. Ghalae *et al*. reported a statistically significant increase in renal colic diagnoses during full moon while Arampatzis *et al*. reported otherwise [15,16]. Our study examines the association between both lunar phases and supermoon events and renal colic incidence.

## Materials and Methods

### Patient Data

Medical records were obtained with a primary diagnosis of urinary calculus based on patient history, physical examination, and confirmation by imaging studies using either non-contrast enhanced spiral abdominal computed tomography or abdominal sonography from the ED at the University of Nebraska Medical Center starting August 16^th^, 2012 and ending July 12^th^, 2014; which are the beginning and ending dates of the lunar months in the 24-month period. The ICD-9 codes used to obtain the data were: 592.0 calculus of kidney; 592.1 calculus of ureter; 592.9 urinary calculus unspecified; 594.0 calculus in diverticulum of bladder; 594.1 other calculus in bladder; 594.2 calculus in urethra; 594.8 other lower urinary tract calculus; 594.9 calculus of lower urinary tract unspecified; 788.0 renal colic. All procedures performed in the current study involving human participants and requirement for informed consent were approved by the IRB at the University of Nebraska Medical Center. More specifically, a waiver for informed consent was granted, as this is a retrospective study so data collection can be performed significantly more quickly as to obtaining informed consent and collecting data over the 24-month period.

### Moon Phase and Supermoon Event Dates

The lunar phases cycle as the moon orbits earth, altering its relative position between earth and the sun, resulting in changes of the lit surface as observed from earth. When the moon appears completely illuminated, it is termed full moon; when the moon is not illuminated, it is termed new moon. The lunar phases are defined as full moon ± two days, new moon ± two days, and the days in-between as normal days according to the lunar calendar.

The combination of the elliptical shape of the moon’s orbit around earth and earth not located in the center of the moon’s orbit alters the distance between the moon and earth. During perigee, or supermoon, the moon is 26,475 miles closer to earth than during apogee, or micromoon. This change in relative position contributes to the supermoon phenomenon appearing 14% larger and 30% brighter as viewed from earth and results in higher tides [17].

### Data Analysis

The dates of the ED diagnoses for renal colic due to ureteral calculus were compared against the lunar phases as well as supermoon events during the 24-month period. The dates of diagnoses confirmed by imaging studies using either non-contrast enhanced spiral abdominal computed tomography or abdominal sonography were grouped into lunar calendar months where the beginning of the month is defined as the beginning of new moon, as opposed to the conventional Gregorian calendar. The number of ED diagnoses for renal colic during different lunar phases and supermoon months were recorded and both Chi-Square and Student’s t-test were performed.

### Power Calculation

Power calculation was performed as previously described [18]. In short, minimal sample size = [Z_1-α/2_^2^ x p x (1 –p)] / d^2^, where Z_1-α/2_ is standard normal variate at *p* = 0.05; p is expected change based on pilot study; d is absolute error or precision. Since Ghalae *et al*. is the only study to date to find a significant difference between full moon (65) and new moon (25.5) ED diagnoses on renal colic, their result of a 60.8% decrease in diagnoses during new moon compared to full moon was used in the calculation. Minimal sample size = (1.96^2^ x 0.608 x 0.392) / 0.05^2^ = 366, and our sample size was 559.

## Results

**Fig 1:**
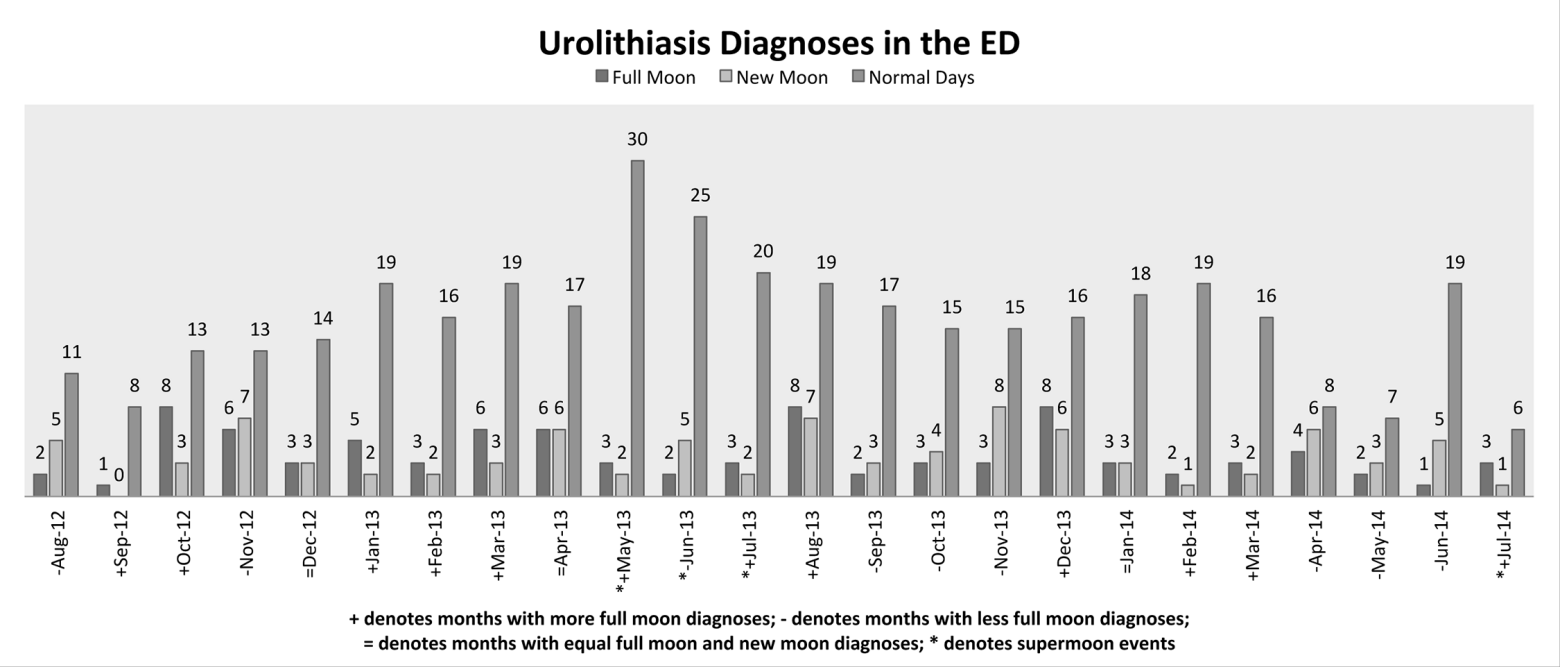
Renal Colic Diagnoses in the ED by Lunar Month Over a 24-Month Period. 559 renal colic cases due to ureteral calculus were diagnosed in the emergency department at the University of Nebraska Medical Center over a 24-month period. 90 cases (16.1%) were diagnosed during full-moon, 89 cases (15.9%) were diagnosed during new moon, and 380 cases (68.0%) were diagnosed during normal days. Supermoon events denoted by *.

559 renal colic patients were diagnosed in the ED of the University of Nebraska Medical Center between August 2012 and July 2014 (0.77 case per day) with primary diagnosis of ureteral calculus based on patient history, physical examination, and confirmation by imaging studies. The patients consisted of 293 females (52.4%) and 266 males (47.6%) with an average age of 52.3 years old. The season with the most urolithiasis diagnoses was summer (144 cases) and the season with the least urolithiasis diagnoses was autumn (129 cases). In total, 90 cases (16.1%) were diagnosed during full moon phase, 89 cases (15.9%) were diagnosed during new moon phase, and 380 cases (68.0%) were diagnosed during normal days. No statistically significant associations were found between increased renal colic ED diagnoses during full moon compared to new moon in this period, *X*^2^ (2) = 0.8187; (*p* = 0.47). During the same period, there were two supermoon events lasting a total of four months (May to July 2013 and July 2014) and no statistically significant associations were found between increased renal colic ED diagnoses during supermoon months compared to the rest of the months, *X*^2^ (1) = 0.2047; (*p* = 0.40), (Fig 1).

## Discussion

In our study with adequate power, the observed seasonal variation in renal colic ED diagnoses due to ureteral calculus could be contributed to volume depletion and dehydration from increased temperature and activity level. This seasonal variance, albeit not statistically significant, correlates with previous published studies [19,20]. In addition, the distribution of diagnoses appeared to be due to chance rather than due to statistically significant associations with lunar phases or supermoon events. The full moon and new moon periods were defined as the day for full moon or new moon ± two days, thus each period lasts five days out of 29.6 days of a lunar month, or 16.9%. Of the 559 diagnoses, 16.1% were during full moon periods and 15.9% were during new moon periods. Chi-square goodness of fit tests were performed and both full moon and new moon diagnoses were distributed due to chance (*p = 1*.*00)*.

Even though our study has adequate power, it is not without limitations. Even though the number of radiographically confirmed diagnoses of 559 had adequate power, this was a retrospective study from a single hospital ED in a 24-month period. In addition, the patient’s stone history, stone burden, and co-morbidities were not used to classify the population as the purpose of the study is to examine the association between lunar phases and ED diagnoses of renal colic due to all causes of ureteral calculus. Furthermore, a control group of patients with renal colic who did not visit the ED could be used to assess true urolithiasis-related ED visits; however, such a control group would not be possible to find with a retrospective longitudinal study.

## Conclusion

Although many studies have found statistically significant associations between lunar phases and changes in human physiology, our findings strongly support that neither full moon phase nor supermoon event is an environmental factor responsible for increased ED visits for renal colic due to ureteral calculus.

## Supporting Information

S1 Table Renal Colic Diagnoses by Moon Phases. (XLSX)
